# Metabolizable Energy of Whole and Ground Canola Seed with Enzyme Supplementation and Effects of Ground Canola Seed on Broiler Performance

**DOI:** 10.3390/ani15223291

**Published:** 2025-11-14

**Authors:** Cleverson de Souza, Ricardo Vianna Nunes, Cleison de Souza, Paula Horácio Cesar, Francieli Sordi Lovatto, Aline Felix Schneider Bedin, Marcelo Suzuki Suyama, Clóvis Eliseu Gewehr

**Affiliations:** 1 Department of Animal Production, Center of Sciences Agroveterinarias of University of Santa Catarina State (UDESC–CAV), Lages 88520-000, SC, Brazil; cleversonsz@hotmail.com (C.d.S.); paulahcesar@gmail.com (P.H.C.); francisordi@hotmail.com (F.S.L.); clovis.gewehr@udesc.br (C.E.G.); 2 Department of Animal Science, State University of Western Paraná (UNIOESTE), Marechal Cândido Rondon 85960-000, PR, Brazil; nunesrv@hotmail.com (R.V.N.); cleisondsz@hotmail.com (C.d.S.); 3 Department of Biosciences and One Health, Federal University of Santa Catarina (UFSC), Curitibanos 89520-000, SC, Brazil; aline.f.schneider@ufsc.br; 4 Department of Animal Science, School of Agricultural and Veterinary Sciences, São Paulo State University (UNESP), Jaboticabal 14884-900, SP, Brazil

**Keywords:** alternative ingredient, biochemical parameters, carcass yield, full-fat canola seed, nutrient digestibility, organ index

## Abstract

**Simple Summary:**

Canola seed is a high-energy oilseed that can be used in broiler diets as an alternative to traditional ingredients, like corn and soybean meal. However, its nutrients are encapsulated by a fibrous hull, limiting digestibility. This study evaluated the effects of grinding canola seed and adding enzymes to improve its nutritional value. The results showed that ground canola seed (GCS) significantly increased energy availability and fat digestibility. Broilers fed up to 150 g/kg of GCS maintained normal growth, metabolism, and carcass traits. Higher inclusion levels reduced performance, especially in early growth. Therefore, GCS can be safely included in broiler diets up to 150 g/kg without adverse effects.

**Abstract:**

Canola seed is a high-energy oilseed with potential as an alternative feed ingredient in broiler diets, yet its use is limited by nutrient encapsulation and antinutritional factors. This study aimed to evaluate the apparent metabolizable energy (AME) of canola seed and its effects on broiler performance, organ development, serum biochemical profile, and carcass yield from hatch to 42 days of age. A digestibility trial was conducted using 300 broilers in a 2 × 2 × 2 factorial design (whole vs. ground seed, with or without enzyme, at 100 or 200 g/kg replacement). A performance trial involved 660 broilers fed diets with 0, 50, 100, 150, 200, and 250 g/kg of ground canola seed (GCS). Grinding without enzyme addition significantly increased AME from 2318 to 3864 kcal/kg and AME corrected for nitrogen retention (AMEn) from 2192 to 3734 kcal/kg (*p* < 0.05). An interaction was observed between seed x enzyme and seed × levels (*p* < 0.05). Addition of enzymes increased the AME value of canola from 3091 to 4091 kcal/kg and the AMEn value from 2963 to 3958 kcal/kg (*p* < 0.001). Inclusion of GCS up to 150 g/kg did not affect feed intake, feed conversion ratio (FCR), organ index, serum parameters, or carcass traits (*p* > 0.05). However, higher inclusion levels (200 and 250 g/kg) reduced body weight (BW) and body weight gain (BWG) and worsened FCR, particularly in the early growth stages (*p* > 0.05). In the period 1–42 days of age, only the 250 g/kg level worsened FCR (*p* < 0.001), and BWG decreased in 200 and 250 g/kg (*p* < 0.001). In conclusion, GCS can be included up to 150 g/kg in broiler diets without compromising performance or metabolism, and grinding combined with enzyme supplementation enhances its nutritional value.

## 1. Introduction

The use of oilseeds in animal nutrition provides potential sources of energy and protein for diet formulations. The canola seed has high levels of ether extract (EE), ranging from 408 to 479 g/kg, giving the seed a high gross energy (GE) value of up to 7126 kcal/kg, and crude protein ranges from 172 to 241 g/kg [1]. Due to its high GE content, canola seed can contribute more AME than canola meal to the diet of poultry. Additionally, canola seed has been shown to have high levels of polyunsaturated fatty acids, mainly oleic acid and linoleic acid, and low concentrations of saturated fatty acids, which make canola an excellent source of lipids in the diet of broilers [2,3,4].

These characteristics allow canola seed to be a substitute for corn and soybean meal, which are commonly used as the energy source and protein in broiler diets, respectively. The use of canola seed in the diet allows greater resistance to oxidation during storage and handling in feed mills when compared to other sources of fat, like vegetable oils [5]. However, canola seed has a hull encapsulating the nutrients inside the seed, so the grinding process improves energy utilization by breaking the hull formed mainly by non-starch polysaccharides (NSPs) [6]. The encapsulation of the oil inside the cell is one of the main problems preventing its use in the feeding of broilers because it reduces the energetic potential of the seed. However, the use of physical treatments and the addition of enzymes to the diet make it possible to improve this potential, as the use of appropriate enzymes can increase the nutritive value of oil seeds for birds [7].

Chickens do not have enzymes to digest the NSPs in the cell wall [8], which limits the use of encapsulated nutrients from the whole canola seed. Incomplete breakage during processing may reduce the utilization and nutritional value of canola [1]. Canola also contains antinutritional compounds, such as glucosinolates and erucic acid, that may limit the inclusion of the seed in chicken diets [4,9,10].

Regarding the energy provided by canola, few studies have been performed analyzing the AME of canola seed. For quails, the AME of the seed ground is 4776 kcal/kg [11]. For ostriches, the true AME for canola grounding is around 5370 kcal/kg [12]. The AME of canola seed for broilers is 2800 kcal/kg [13], and for canola seed steam pelleted, the value is 4664 kcal/kg [1].

According to Rutkowski et al., seed grinding improves performance, AME, and digestibility of canola for broiler chickens [13]. These benefits are observed due to the greater availability of protein and EE for digestion. Although previous studies have reported the feasibility of using canola seed, there are no studies using GCS in broiler diets with high inclusions without enzymatic or heat treatment and from hatch to 42 days of age, evaluating the effects on productive performance, organ weight, serum biochemical profile, and carcass yield. We hypothesize that grinding the canola seed will increase the energy value; the use of the enzymatic blend will improve the energy utilization of the canola seed; and the canola seed can be included in the broiler diet only with the grinding process up to a certain level, without adversely affecting growth performance from hatch to 42 days of age.

Therefore, the objective was to evaluate the digestibility of canola for broilers in whole and ground seed, with and without the utilization of an enzymatic blend. Additionally, the objective was to evaluate the effects of the inclusion of GCS in the diet of broilers, from 1 to 42 days old, on the performance, organ weight, serum biochemical profile, and carcass yield.

## 2. Materials and Methods

### 2.1. Ethics Statement

The experiment was conducted in Lages, SC, Brazil, at a latitude of −27.8167° S and a longitude of −50.3264° W and an elevation of 930 m, in accordance with the regulations issued by the National Council for Animal Experimentation and approved by the Ethics Committee on Animal Use of the State University of Santa Catarina under approval number 27262701162.

### 2.2. Canola Seed

The canola seed used was hybrid Hyola 76 (Advanta Seeds, Staufen, Germany), and it was used in the diets of broiler chickens, with a chemical composition of 950 g/kg dry matter (DM), 46 g/kg ash, 238 g/kg crude protein (CP), 295 g/kg EE, 171 g/kg neutral detergent fiber (NDF), 11.7 g/kg acid detergent fiber (ADF), and 6385 kcal/kg of GE on a DM basis. The canola was ground in a hammer mill, using a 4.5 mm sieve, for inclusion in the experimental diets.

### 2.3. Experimental Design and Treatments

Male Cobb 500 broilers were raised until the beginning of the digestibility trial and received an initial diet formulated to meet nutritional requirements, according to Rostagno et al. [14]. At 17 days of age, 300 broilers were allocated in metabolism cages, measuring 50 cm × 50 cm in an air-conditioned room and distributed in a completely randomized design in a 2 × 2 × 2 factorial arrangement (replacement level 100 or 200 g/kg x; physical form seed or ground seed × enzyme blend, with or without), and they were fed a reference diet (RD) with or without the enzyme blend. There were 10 treatments in total; each treatment had five replications with six birds each.

Eight test diets with canola were provided, and for each treatment, the RD was replaced by 100 or 200 g/kg with whole canola seed (WCS) or GCS. The RD was formulated based on corn and soybean meal (Table 1), considering the nutritional requirements of the broilers according to Rostagno et al. [14].

The enzyme blend used in this experiment was composed of carbohydrases, proteases, and phytase obtained from the fermentation of Aspergillus niger and Trichoderma reesei fungi (Poultrygrow, Safeeds Nutrição Animal, Cascavel, PR, Brazil) with the following composition: Endo-1,4-Beta-Xylanase 300 U/g (IUB/EC 3.2.1.8); 6-Phytase 1000 FTU/g (IUB/EC 3.1.3.26); Protease 6250 U/g (IUB/EC 3.4.21), Vitamin A 2000 UI/kg; Vitamin D3 600 UI/kg; and Vitamin E 15 UI/kg. The enzyme was added on top at a concentration of 150 g per ton of feed. During the experimental phase, the birds received feed in mash form and water ad libitum. The geometric mean diameter (GMD) and geometric standard deviation of the test diets and canola (Table 2) were determined using the fixed method using sieves with openings of 4000, 2000, 1190, 595, 297, and 149 μm; the calculations were performed using Granucalc software (Embrapa Suínos e Aves, Desktop version 2013, Concórdia, SC, Brazil) [15].

### 2.4. Sample Collection and Analysis

The experimental period consisted of eight days, with four days of adaptation (17 to 21 days of age) and four days of excreta collection (21 to 25 days of age). To mark the beginning and end of the collection period, 10 g/kg of ferric oxide was used in the diet, and at the end of the period, FI and total excreta production were determined during excreta collection. The excreta were collected in trays previously coated with plastic to avoid contamination and losses. The samples were collected twice a day, at intervals of 12 h, to avoid possible fermentation that could alter the composition of the samples.

The excreta of each collection were stored in plastic bags properly identified and conditioned in a freezer at −20 °C for later laboratory analysis. After thawing and homogenizing, a 300 g sample of each replicate was oven-dried at 55 °C for 72 h to promote pre-drying and to perform the determination of dry matter in air. Then, the samples were ground in a knife-type mill, with a 16-mesh sieve and a 1 mm sieve. After that, the samples were analyzed following the methodology described by AOAC [16] to determine DM (AOAC: method 930.15). Nitrogen was determined by the combustion method using the Leco AC500 analyzer (Leco Corp., St. Joseph, MI, USA). The CP was obtained by multiplying the N content by 6.25. EE was determined through extraction with petroleum ether in the Soxhlet extractor (AOAC: method 920.39), and GE was determined using a calorimetric bomb (IKA C200, IKA-Werke GmbH & Co., Staufen, Germany). The analysis of NDF and ADF was determined using the ANKOM200 fiber analyzer (ANKOM Technology, Macedon, NY, USA) (AOAC: method 973.18) and was performed on canola seed.

### 2.5. Calculations of Metabolizable Energy and Metabolizable Coefficients

The values of AME and AMEn were calculated using equations obtained from the chemical composition of experimental diets, excreta, and canola seed. Equation (1), proposed by [17] is used to calculate AME as follows:

(1) $$AME\;(kcal/kg,\;DM)=\left(GEing-GEexcr\right)÷DMing$$

where GEing is the product of the dry matter ingested (DMing) by the GE of the diet and GEexcr is the product of the amount of excreta produced by the GE of the excreta. The AMEn value was calculated utilizing a correction factor of 8.22 kcal/kg of retained nitrogen [18]. To calculate the AME of the canola, Equation (2) was used as follows:

(2) $$AMEcs\;(kcal/kg,\;DM)={AME}_{rd}+\left[\left({AME}_{td}-{AME}_{rd}\right)÷Pcs\right]$$

AME_rd_ is the AME of RD and AME_td_ is the AME of diet with canola substitution. P_CS_ was the replacement level (%) of canola corrected for DM. The metabolizable coefficients of dry matter (MCDM), crude protein (MCCP), ether extract (MCEE), AME (MCAME), and AMEn (MCAMEn) were calculated based on the chemical values of canola and the results obtained in the digestibility trial.

### 2.6. Experimental Design, Treatments, and Animals

A total of 660 one-day-old male Cobb 500 broilers were distributed in a completely randomized design with six levels of inclusion of ground canola seed in the diet (0, 50, 100, 150, 200, and 250 g/kg), totaling six treatments with five replicates of 22 birds each. The GMD of canola used in the experiment was 955 μm after the grain was grounded. The chemical composition of canola was used in the formulation of experimental diets and with an AME of 3864 kcal/kg. The canola used did not undergo any thermal treatment to deactivate the possible antinutritional factors.

The feeding program was divided into four phases: pre-initial (1 to 7 days), initial (8 to 21 days), growth (22 to 33 days), and final (34 to 42 days). The rations were formulated using the values of chemical composition and nutritional requirements for broilers recommended by [14] for all ingredients, except for canola seed, whose values were determined by bromatological analysis. The experimental diets and their calculated composition for each phase are presented in Table 3 (pre-initial and initial) and Table 4 (growth and final).

The birds were housed in a ventilated, temperature-controlled shed, with ad libitum access to water and feed in mash form. The temperature was maintained at 32 °C during the first 3 days of age. Thereafter, it was gradually lowered by approximately 3 °C per week until reaching 25 °C by the end of the third week. Light intensity was maintained at 30–40 lux during the experimental period. Artificial lighting was continuously provided for the first 24 h and then gradually reduced until reaching 18 h at the end of the second week.

### 2.7. Data Collection

The feed intake (FI) and the body weight (BW) were recorded weekly by repetition (days 7, 14, 21, 28, 35, and 42). The body weight gain (BWG) and feed conversion ratio (FCR) were calculated from these data and corrected for mortality.

At 42 days of age, two birds per replicate were randomly selected to collect 5 mL of blood from the jugular vein. The blood was immediately transferred to a microtube without an anticoagulant. The samples were left at rest for 40 min. Then, they were centrifuged at 3500 rpm for 10 min. The collected serum was stored in 2 mL plastic tubes and kept at −20 °C for further analysis. Serum levels of uric acid (uricase method), total proteins (biuret method and refractometry), total serum calcium (colorimetric method), chlorides (colorimetric method), cholesterol (enzymatic hydrolysis and oxidation), alkaline phosphatase (colorimetric method), total serum phosphorus (colorimetric method), and triglycerides (glycerol–phosphate oxidase method) were determined in duplicate and performed by spectrophotometry using an automatic biochemistry analyzer (Labmax Plenno, Labtest Diagnóstica S.A., Lagoa Santa, MG, Brazil). The reagents used were from commercial kits, utilized according to the manufacturer’s guidelines (Labtest Diagnóstica S.A., Lagoa Santa, MG, Brazil).

At the end of the trial, at 42 days of age, two birds per experimental pen were selected based on average weight and subjected to a 24 h fast. They were stunned by electroshock and sacrificed through cervical dislocation to obtain organ and digestive tract weights, carcass weights, and commercial piece cut-up weights. These weights were then used for the calculation of the organ index, carcass yield, and piece cut-up yield. The gizzard index, liver index, spleen index, heart index, and intestine index are calculated in relation to the carcass weight. For analysis of carcass yield, the weight of the carcass without feet, head, and abdominal fat was considered in relation to the live weight of the bird. For the calculation of the yields of piece cut-ups, the breast, thigh and overcoat, wing, and back were considered, along with skin and bones. These yields were calculated in relation to the weight of the eviscerated carcass.

### 2.8. Statistical Analyses

The data were statistically analyzed using the GLM procedure with SAS statistical software (Version 9.2, SAS Institute Inc., Cary, NC, USA), considering a significance level of *p* < 0.05. The model used to describe the data in the digestibility trial is as follows:

Yij = µ + Ti + Ej + Ti × Ej + eij(3)

where Yij is the response variable, μ is the general average, Ti is the treatment used (i = whole seed or ground), Ej is the addition of the commercial enzyme (j = yes or no), Ti × Ej is the interaction between the treatment used (whole seed or ground) and addition of enzyme (yes or no), and eij is the experimental error. The means were compared using Tukey’s HSD test.

The data in the performance trial were analyzed using analysis of variance (ANOVA). When there were significant observations, the data were further analyzed by polynomial regression to determine whether the effect of the treatments was linear or quadratic. Significant data that did not fit polynomial regression were analyzed using Dunnett’s test to compare treatment means to the control (0 g/kg). The statistical model adopted is as follows:

Yij = µ + Ti + eij(4)

where Yij is the observed value of the studied variable j subjected to treatment i; µ is the overall mean of all observations; Ti is the effect of the treatment (i = 0, 50, 100, 150, 200, and 250 g/kg); and eij is the experimental error.

## 3. Results

### 3.1. Digestibility Trial

The grinding process increased the AME and AMEn levels of the canola from 3000 to 4182 and from 2872 to 4049 kcal/kg, respectively (Table 5). The MCEE was influenced (*p* < 0.001) only by grinding the seed, raising the coefficient from 56.9 to 72.9%. Grinding increased (*p* < 0.001) the MCAME and MCAMEn from 46.9 to 65.5% and from 44.9 to 63.4%, respectively, when compared to the whole canola seed. The MCDM and MCCP were not influenced by grinding, enzyme, or level (*p* > 0.05).

The addition of enzymes (Table 5) increased the AME value of canola from 3091 to 4091 kcal/kg and the AMEn value from 2963 to 3958 kcal/kg (*p* < 0.001). The addition of the enzymes increased from the MCAME from 48.4 to 64.1% and the MCAMEn from 46.4 to 61.9% in relation to the absence of enzymes. The addition of enzymes did not influence (*p* > 0.05) the MCEE (*p* = 0.080).

The substitution level influenced the AME, AMEn, MCAME, MCAMEn, and MCEE, with the 100 g/kg substitution level presenting higher values for these variables compared to the 200 g/kg substitution level (*p* < 0.05).

An interaction was observed between the treatments (Table 6) for the MCAME and the MCAMEn; it was observed that, regardless of the use of the enzyme, grinding the seed increased the value of the analyzed variables. In the absence of the enzyme, grounded canola seed increases the AME from 2318 to 3864 kcal/kg and the AMEn from 2231 to 3734 kcal/kg, while the MCAME increased from 36.3 to 60.5% and the MCAMEn from 34.9 to 58.5%. When using the enzyme, the grinding process increased AME from 3906 to 4500 and AMEn from 3552 to 4363 kcal/kg, while the MCAME increased from 61.2 to 70.5% and the MCAMEn went from 59.3 to 67.4%.

An interaction between the physical treatment and the canola substitution level was also observed for the values of the AME, AMEn, MCAME, and MCAMEn (Table 6). The 100 g/kg inclusion level presented higher values of these variables forWCS. However, the substitution level (100 or 200 g/kg) did not alter the values of AME, AMEn, or their metabolizable coefficients for GCS.

The MCAME and MCAMEn in the experimental diets were analyzed as a function of the treatments (Table 7). It was observed that the addition of the GCS showed higher coefficients than the addition of the WCS to the diet (*p* < 0.001). The same was observed for the diets containing the enzyme blend compared to those without the enzyme (*p* = 0.001).

### 3.2. Performance Trial

There were no linear or quadratic trends (*p* > 0.05) based on the inclusion of GCS levels in the diet growth performance (Table 8). Regarding the mean weekly BW of the birds, there was a significant effect from the inclusion of canola on the mean BW in all the evaluated weeks (*p* < 0.05), where birds that consumed 200 and 250 g/kg of GCS in the diet had lower weights at 7, 14, 21, 28, 35, and 42 days when compared to the control. In addition, it was observed that in the first and second week of age, the inclusion of 150 g/kg of canola in the diet reduced the mean BW (*p* = 0.005); however, this was not observed in subsequent weeks.

Regarding BWG, it was observed that in the first week of age, the inclusion of 150, 200, and 250 g/kg of GCS in the diet reduced BWG compared to the birds that consumed a diet without GCS (*p* < 0.05). In the second and third weeks, only the birds that received 250 g/kg of GCS in the diet showed lower BWG than those fed with 0 g/kg (*p* < 0.05). For the other weeks, no effect of canola inclusion on the BWG of the birds was observed (*p* > 0.05). In the period 1–42 days of age, BWG at the 200 and 250 g/kg levels differed from the 0 g/kg level (*p* < 0.001). The inclusion of GCS in the diet did not alter the FI of the birds in any of the evaluated time points (*p* > 0.05) (Table 7). For FCR, it was observed that in the first week of age, birds receiving 200 and 250 g/kg of GCS in the diet presented worse FCR compared to the birds that received 0 g/kg (*p* < 0.05). In the other weeks, there were no statistical differences for bird FCR between treatments (*p* > 0.05) (Table 8). From 1 to 42 days of age, FCR at 250 g/kg GCS inclusion was worse than at the 0 g/kg level (*p* < 0.001).

The carcass yields and piece cut evaluation showed no effect of treatments for carcass yield, breast, thigh and upper leg, wing, and back (*p* > 0.05) (Table 9).

No effects of GCS in the diet were observed on uric acid, total protein, Ca, chloride, cholesterol, alkaline phosphatase, phosphorus, and triglycerides serum levels (*p* > 0.05) (Table 10).

The evaluation of the organ index, as presented in Table 11, did not present a significant difference (*p* > 0.05) based on the levels of inclusion of GCS in the diet on the indices of the gizzard, liver, spleen, heart, and intestine.

## 4. Discussion

### 4.1. Digestibility and Energy Value of Whole and Ground Canola Seed With or Without Enzymes

Canola (Canadian Oil Low Acid) is the result of the genetic improvement of rapeseed (*Brassica napus* L.) and has a lower erucic acid content in the oil (<20 g/kg) and glucosinolates in the grain (7.4 µmol/g), improving the palatability and digestibility of nutrients compared to rapeseed [2]. The use of canola seed in the diet of broiler chickens is of great interest to researchers and animal nutritionists due to the high EE content of 295 g/kg; the canola used has a high GE content of 6385 kcal/kg. It can be an alternative energy source in the diet of broiler chickens.

The main problem with using canola seed is the encapsulation of its nutrients by the hull, which is a limiting factor for the use of its nutrients by non-ruminants since these animals do not have the ability to digest cell wall NSPs [5,19]. However, the application of physical treatments can improve the use of these encapsulated nutrients, as observed in this study, since there was an increase of 39.4% in AME, 40.9% in AMEn, and 28.1% in MCEE when the grain was subjected to the physical treatment of grinding.

Ref. [13] observed an increase in nitrogen retention, total fat digestibility, and AME, where nitrogen retention increased from 50.8 to 55.7%, total fat digestibility increased from 33.3 to 64.0%, and AME increased from 2652 to 2818 kcal/kg, representing an increase of 9.6, 47.9, and 9.1%, respectively, when the canola seed underwent the grinding process. The reduction in particle size improves the action of digestive enzymes by breaking the hull and reducing the size of the particles, enhancing the utilization of nutrients and energy from the canola seed. The seed without the grinding process showed a GMD of 1570 μm, while after grinding, the GMD was 955 μm.

The energetic value of canola was determined using two substitution levels to obtain the AME values through the regression method. However, there was no statistical difference in the AME value between the substitution levels of GCS, so the value obtained of 3864 kcal/kg refers to the pool of 100 and 200 g/kg without the enzyme, and with the enzyme, the value was 4500 kcal/kg. The result obtained indicates that a single substitution level can be used for the evaluation of fat-rich feedstuffs for birds. The regression method becomes more appropriate for the evaluation of fiber-rich feedstuffs, as digestibility decreases linearly with the increase in NDF and ADF content in diets [20].

Canola has an average NDF and ADF content of 292 g/kg and 221 g/kg, respectively [1]. However, in our study, the values of NDF and ADF were 171 g/kg and 117 g/kg, respectively, which reduced the fiber concentration in the test diets and did not compromise the digestibility of the diets. Nonetheless, the WCS exhibited a significant difference between the replacement levels, potentially attributable to the intact hull of the canola seed, which compromised the digestibility of the diets compared to the GCS.

Barbour et al. obtained values of true AME and true AMEn of 4623 and 4487 kcal/kg, respectively, for WCS for roosters [21]. In the present study, only the AME and AMEn were measured, and, therefore, the values found are lower than those observed by the authors and by Brand et al., who evaluated the true AME of WCS for ostriches with a value of 5374 kcal/kg [12]. This decrease in the value of AME for broilers may be related to the smaller size of the digestive tract compared to other species [22], reducing the utilization of the energy from canola seed.

Although the grinding process and enzyme addition increased the energy value of canola seed, no effect of the treatments on the MCCP was observed. It is known that antinutritional properties that occur naturally in leguminous and canola seeds may impair protein digestion and amino acid uptake by poultry. Among these components, we can mention substances such as tannin and trypsin inhibitors in vegetables, phytase, and non-amylaceous polysaccharides in canola [10]. These components are present in raw material in different quantities, depending on the species, variety, region, meteorological conditions, and other factors [1]. Although antinutritional factors were not measured in this work, grinding and the addition of enzymes did not alter protein digestibility.

Despite the energy value of GCS being higher than WCS, the MCDM did not differ with the grinding process, the enzyme, and the substitution level. It was expected that the MCDM of WCS would be lower due to the encapsulation of the canola seed, but it did not differ from GCS. However, the MCEE of GCS was higher than that of WCS (72.9 vs. 56.9%); consequently, the energy utilization of ground canola was higher. The enzyme did not show an effect on the MCDM, so the use of the enzyme at recommended levels may not be sufficient to degrade the β-bonds of the NSP into simple sugars and increase the digestibility of DM [20]. Nonetheless, the enzyme blend employed effectively mitigated the antinutritional impacts of NSPs and enhanced the digestibility of energy and EE.

With the use of enzymes in diets with canola for broiler chickens, Meng et al. found a significant increase in digestibility in fat, NSPs, and AMEn when compared to the control diet [5]. The addition of cellulase, pectinase, xylanase, glucanase, and mannanase together increased fat digestibility from 63.5% to 82.4%, the digestibility of NSPs from 4.4 to 25.8%, and the metabolizable energy values from 3642 to 4869 kcal/kg. [13] tested whether grinding and enzyme addition could increase the energy value of canola for broilers. With the enzyme, there was an increase in nitrogen retention, where the value increased from 51 to 55.4%, and EE digestibility increased from 45.6 to 51.7% and AME from 2698 to 2771 kcal/kg, corroborating the data observed in the present study, where there was an increase in the digestibility of EE and energy of canola with the addition of enzymes.

The main way to include canola in the diet of broiler chickens is with canola meal. In a study developed by [23], the canola meal used had 10.5 g/kg of EE, 4143 kcal/kg of GE, and a high concentration of CP with 408.8 g/kg in DM. The authors obtained values of 2286 and 1931 kcal/kg of AME and AMEn, respectively, in DM. The GCS showed values of 3864 and 3734 kcal/kg of AME and AMEn, respectively, in DM. This indicates that canola seed has a higher AME than canola meal, making it an excellent source of energy and having about half the concentration of CP compared to canola meal.

### 4.2. Effects of the Inclusion of Ground Canola Seed in the Diet of Broilers

Most studies do not use canola seed in the diet in its integral form without thermal treatment [1,9,19,24,25], enzymatic treatment [19,24], or even radiation treatment [10] for broilers. The AME values of 3864 kcal/kg, derived from the digestibility trial of GCS without enzyme utilization, were used in the formulation of the experimental diets for the performance trial to evaluate the effect of high inclusions of canola seeds only on the grinding process.

In a recent study, [4] included canola seeds in the diet of chickens, but in low concentration with 50 g/kg in the pre-initial phase, 60 g/kg in the initial phase, 80 g/kg in the growth phase, and 100 g/kg in the final phase, and there was a reduction in FI. It is possible that the seed presents the presence of residual isothiocyanate from the breakdown of glucosinolates by the enzyme myrosinase; isothiocyanate can alter the palatability of the diets, potentially reducing feed intake due to their bitter taste [26]. Thus, diets with canola seed tend to have less palatability, which reduces FI by birds and, consequently, their performance [9].

However, in this study, it was demonstrated that there was no effect of the inclusion of GCS up to 250 g/kg in the diet on the FI, even when used without thermal or enzymatic treatments. It is known that the composition and content of glucosinolates in the grain vary due to species, cultivation practices, and climatic conditions, among others, and that plants grown under tropical climates have more of these compounds than plants grown in temperate climates [26].

Thus, as the canola used in the experiment came from Rio Grande do Sul, Brazil, which has a temperate climate, it may present a lower amount of glucosinolates, which would justify the absence of effect on consumption because glucosinolate concentrations in the diet from canola are negatively related to feed intake in broiler chickens [4]. This reduction in the glucosinolate content could be measured through the activity of the enzyme myrosinase. However, under the conditions of this experiment, its measurement was not feasible since the enzyme acts rapidly after grinding the seed [25].

Although FI was not affected, GCS over 150 g/kg in the diet reduced the BW and BWG of birds in the first and second week of age. From after the second week of age, only the inclusion levels above 200 g/kg caused losses in performance. It is well known that canola seed has antinutritional factors such as glucosinolates, derived toxic metabolites, phytate, tannins, sinapine, and erucic acid of canola that reduce the performance of the birds [2,4,26].

Additionally, the increase in the level of inclusion of GCS increases the amount of dietary fiber in the gastrointestinal tract (GIT) of birds, which reduces the digestibility and absorption of nutrients [20]. In the digestibility trial, no statistical difference was found in the AME of the diets between the levels of 100 and 200 g/kg. However, the trial lasted eight days, while in the performance trial, the birds received GCS from the first day of age. Adult birds have a superior capacity to obtain energy from feed compared to young birds due to the development of the GIT, which can adapt to improve the digestion of dietary fiber derived from the canola hull [27]. The chickens consumed canola from the first day after hatching, so the GIT underwent adaptation to digest the NSPs. However, a reduction in BWG was observed at levels of 200 and 250 g/kg, and FCR worsened at the level of 250 g/kg, in the evaluation of the period from 1 to 42 days of age, which can be attributed to the manifestation of its antinutritional effects due to the increased intake of dietary fibers, and the antinutritional factors may have impaired the utilization of nutrients in the feed.

In addition, the production of chymotrypsin and trypsin increases with the passage of bird age, with the maximum values of these enzymes being reached at 15 days of age [28]. This may lead to a protein underutilization of GCS. This protein and lipid underutilization can reduce the metabolizable energy of growing broiler diets, as reported by Barekatain et al., who also observed a reduction in the digestibility coefficients of methionine, leucine, threonine, alanine, glutamine, and proline present in canola seed diets when compared to a diet containing canola meal [25].

The low performance of broilers with the inclusion of canola in the diet was also observed by Kandel et al.; the inclusion of GCS from 50 g/kg depressed BWG and reduced FI [4]. However, in a study developed by Hamadi et al., no significant difference was obtained in FCR between diets for broilers with the inclusion of up to 150 g/kg of GCS with electron beam irradiation to reduce tannins and glucosinolates in canola seed [10]. In our study, broilers consumed canola seed from day one, only through the grinding process, and it can be included in the diet at levels of up to 150 g/kg without negative effects on growth performance. These results indicate that the composition of antinutritional components of the canola seed variety, such as glucosinolates and dietary fiber, influences the productive performance of birds.

Regarding the carcass yield and piece cuts, it can be inferred that the canola levels used did not cause a nutritional imbalance capable of altering the absorption or protein deposition in the carcass. It is known that changes in the constituents of the carcass are linked to the absorption of nutrients. In view of this, it can be concluded that canola seeds met the nutritional needs for maintaining carcass conformation without negative effects, even with high inclusions of up to 250 g/kg. The inclusion of GCS in the diet of broilers also had no effect on the carcass in the experiments conducted by Kandel et al., with inclusion of up to 100 g/kg [4], Hamadi et al., with inclusion of up to 150 g/kg with canola reduced in tannins and glucosinolates [10], and Lee et al., with inclusion of up to 200 g/kg [29].

Since no differences were observed in the relative organ weights and serum biochemical profile, it can be stated that the use of canola in diets did not interfere with metabolism, and its nutrients were well utilized by the broiler chickens. It is well known that blood components can be influenced by physiological, pathological, and nutritional factors [30]. In laying Japanese quails fed with GCS up to 150 g/kg, no difference was found in relation to total protein, renal function biomarkers, lipid profile, and liver enzyme activity [2]. However, there was a change in serum triglycerides, and this was due to the high content of EE present in the canola seed used by the authors compared to the present study (489 vs. 295 g/kg).

Regarding the use of GCS in diet in relation to the organ index, Barekatain et al. observed an effect only on the jejunum index when comparing canola seed to canola meal; the other organs analyzed were not influenced [25]. These results are due to the higher fiber composition in the seed than in canola meal. The increase in fiber consumption is related to the development and adaptation of the GIT, especially the gizzard for fiber digestion, leading to an increase in the relative weight of the digestive organs [8]. Relative weights of the GIT and gizzard increased by increasing levels of GCS in the diet, as observed by Hamadi et al. [10]. However, no significant difference was observed in the evaluated levels of the study.

In addition to what was previously discussed, it is interesting to note that our work analyzed the performance of birds consuming GCS from the first day of life and with higher inclusion compared to other studies. This fact may explain the difference in poultry performance in the first weeks, which was reflected in the final weight. Based on this, new work should be performed to determine the best age to include canola in the diet and to determine the optimal inclusion levels.

## 5. Conclusions

The present study demonstrates that the physical processing of canola seed through grinding, combined with the use of an enzymatic blend, significantly enhances its digestibility and energy utilization in broiler diets. GCS showed superior values of AME and EE digestibility compared to WCS, confirming the importance of particle size reduction for nutrient availability. The inclusion of GCS up to 150 g/kg in the diet did not compromise feed intake, organ development, serum biochemical parameters, or carcass yield, indicating its viability as an alternative energy source in broiler nutrition.

However, inclusion levels above 150 g/kg resulted in reduced body weight and weight gain, particularly in the early stages of growth, likely due to the presence of antinutritional factors and increased dietary fiber. These findings suggest that while GCS can be effectively used in broiler diets, its inclusion should be limited to 150 g/kg to avoid performance losses, with only physical treatment through grinding, without enzymatic or thermal treatment. Further studies are recommended to evaluate the optimal age for GCS introduction and to explore strategies for mitigating the effects of antinutritional compounds, thereby maximizing its nutritional potential in poultry production systems.

## Figures and Tables

**Table 1:** Experimental composition of reference diet; as fed-basis.

Ingredients, g/kg	Reference Diet
Corn	617.5
Soybean meal	310.0
Dicalcium phosphate	13.8
Soy oil	35.0
Limestone	8.15
Salt	3.70
L-Lysine	3.35
DL-Methionine	2.5
Adsorbent	2.0
Mineral and vitamin premix ^1^	4.0
Total, kg	1.0
Calculated composition	
AME, kcal/kg	3150
Crude protein, g/kg	198.0
Calcium, g/kg	7.7
Phosphorus available, g/kg	3.63
Digestible Lys, g/kg	11.7
Digestible Met, g/kg	2.0

^1^ Composition of mineral and vitamin premix (levels per kg of product): Vitamin B12—3000 µg, Vitamin B6—622 mg, Pantothenic Acid—2934.9 mg, Niacin—7500 mg, Biotin—19 mg, Vitamin B2—1125 mg, Manganese—16,800 mg, Zinc—13,000 mg, Iron—12,600 mg, iodine—250 mg, Copper—2100 mg, Vitamin E—3650 UI Vitamin K3—450 mg, Vitamin B1—502 mg, Folic Acid—189 mg, Colistin—2.500 mg, BHT—0.80 g, Choline—86.67 g.

**Table 2:** Geometric mean diameter (µm) and geometric standard deviation of diets, whole canola seed, and ground canola seed.

Item	Geometric Mean Diameter	Geometric Standard Deviation
WCS	1570	1.12
GCS	955	1.72
RD + 100 g/kg WCS	580	2.61
RD + 200 g/kg WCS	605	2.13
RD + 100 g/kg GCS	588	2.18
RD + 200 g/kg GCS	608	2.02

GCS—ground canola seed; RD—reference diet; WCS—whole canola seed.

**Table 3:** Nutritional and calculated composition of experimental pre-initial (1 to 7 days of age) and initial (8 to 21 days of age) diets; as fed-basis.

	Levels of Inclusion (g/kg)												
	Pre-Initial (1 to 7 Days of Age)							Initial (8 to 21 Days of Age)					
Ingredients, g/kg	0	50	100	150	200	250		0	50	100	150	200	250
Corn	565.8	538.5	521.0	481.3	440.7	408.0		561.1	515.1	510.0	449.0	438.0	450.0
Soybean meal	356.2	350.5	326.0	300.0	274.2	250.0		343.0	320.0	294.0	290.0	254.0	230.0
Dicalcium phosphate	21.8	35.0	35.3	35.6	35.9	36.0		30.0	30.0	29.9	29.9	30.0	30.0
Soy oil	20.0	9.6	0.3	0.0	0.0	0.0		35.0	38.0	23.0	30.0	20.0	0.0
Wheat bran	12.0	0.0	0.0	14.6	29.7	36.0		16.0	30.0	23.0	30.0	40.0	23.0
Limestone	8.2	0.0	0.0	0.0	0.0	0.0		0.0	0.0	3.0	5.0	0.0	0.0
Salt	5.0	5.0	5.0	5.0	5.0	5.0		4.0	4.5	4.0	4.0	4.0	4.0
L-Lysine	4.0	4.2	5.0	5.9	6.7	7.5		2.8	4.0	4.5	4.3	5.0	5.0
DL-Methionine	2.2	2.2	2.4	2.6	2.8	3.0		3.1	3.4	3.7	4.0	4.0	4.0
Ground canola seed	0.0	50.0	100.0	150.0	200.0	250.0		0.0	50.0	100.0	150.0	200.0	250.0
Mineral and vitamin premix ^1^	5.0	5.0	5.0	5.0	5.0	5.0		5.0	5.0	5.0	5.0	5.0	5.0
Total, kg	1.0	1.0	1.0	1.0	1.0	1.0		1.0	1.0	1.0	1.0	1.0	1.0
Calculated composition													
AME, kcal/kg	2966	2960	2960	2960	2960	2989		3050	3066	3052	3088	3078	3081
Dry matter, kcal/kg	884.4	897.3	898.9	902.4	905.6	901.2		892.4	891.8	891.0	900.4	907.2	902.6
Ether extract, g/kg	46.7	49.7	54.1	67.45	80.5	93.9		61.6	77.6	76.6	96.2	100.4	94.6
Crude protein, g/kg	224.0	224.0	224.0	224.1	224.0	224.0		212.0	213.5	212.9	218.4	214.9	214.5
Calcium, g/kg	9.9	10.0	10.0	10.0	9.9	9.9		8.8	8.7	9.7	10.5	8.5	8.4
Phosphorus available, g/kg	5.2	7.6	7.6	7.6	7.6	7.5		6.7	6.7	6.6	6.6	6.5	6.4
Digestible Lys, g/kg	13.3	13.2	13.2	13.2	13.2	13.2		12.2	12.4	12.2	11.8	11.4	10.8
Digestible Met, g/kg	5.2	5.2	5.2	5.2	5.2	5.1		6.0	6.2	6.3	6.4	6.2	6.1
Digestible Met + Cys	8.2	8.0	7.8	7.7	7.5	7.3		8.9	8.9	8.8	8.9	8.5	8.2
Digestible Try, g/kg	2.5	2.5	2.3	2.1	2.0	1.8		2.5	2.3	2.1	2.1	1.9	1.7
Digestible Ile, g/kg	8.5	8.3	7.8	7.2	6.6	6.1		8.2	7.7	7.2	7.0	6.3	5.7
Digestible Arg, g/kg	13.5	13.2	12.3	11.5	10.6	9.8		13.1	12.4	11.4	11.2	10.1	9.2
Digestible Thr, g/kg	7.5	7.3	6.9	6.4	5.9	5.4		7.3	6.9	6.4	6.2	5.6	5.2
DEB	209	200	181	164	148	130		209	191	172	169	148	131
Neutral detergent fiber, g/kg	129.2	128.8	131.7	137.2	142.7	146.1		128.7	133.5	135.1	137.5	144.1	144.1
Acid detergent fiber, g/kg	48.9	51.9	55.2	59.5	63.9	67.5		48.3	52.6	55.2	59.7	63.6	65.5

AME—apparent metabolizable energy; DEB—dietary electrolyte balance (DEB = K + Na − Cl). ^1^ Composition of mineral and vitamin premix (levels per kg of product): Vitamin B12—3000 µ, Vitamin B6—622 mg, Pantothenic Acid—2934.9 mg, Niacin—7500 mg, Biotin—19 mg, Vitamin B2—1125 mg, Manganese—16,800 mg, Zinc—13,000 mg, Iron—12,600 mg, iodine—250 mg, Copper—2100 mg, Vitamin E—3650 UI Vitamin K3—450 mg, Vitamin B1—502 mg, Folic Acid—189 mg, Colistin—2.500 mg, BHT—0.80 g, Choline—86.67 g.

**Table 4:** Nutritional and calculated composition of experimental growth (22 to 33 days of age) and final (34 to 42 days of age) diets; as fed-basis.

	Levels of Inclusion (g/kg)												
	Growth (22 to 33 Days of Age)							Final (34 to 42 Days of Age)					
Ingredients, g/kg	0	50	100	150	200	250		0	50	100	150	200	250
Corn	586.5	596.5	585.5	566.3	540.0	502.0		668.0	651.0	634.1	617.2	583.0	542.2
Soybean meal	306.6	282.0	258.0	235.0	211.5	182.5		267.7	242.6	217.4	192.2	166.0	139.9
Dicalcium phosphate	19.9	19.9	19.9	19.9	19.9	19.9		19.8	20.2	20.5	20.8	21.2	21.5
Soy oil	42.8	26.6	14.4	5.4	0.0	0.0		30.8	21.4	11.9	2.5	0.0	0.0
Wheat bran	15.7	4.9	0.0	0.0	5.0	22.0		0.0	0.0	0.0	0.0	11.3	26.6
Limestone	4.8	5.0	5.3	5.5	5.7	5.9		0.0	0.0	0.0	0.0	0.0	0.0
Salt	4.0	4.0	4.0	4.0	4.0	4.0		4.0	4.0	4.0	4.0	4.0	4.0
L-Lysine	2.9	3.7	5.0	5.5	6.0	6.5		3.1	4.0	4.9	5.7	6.6	7.5
DL-Methionine	1.8	1.9	2.5	3.0	2.4	2.7		1.6	1.7	1.9	2.1	2.3	2.5
L-Tryptophan	0.0	0.5	0.4	0.4	0.5	0.7		0.0	0.1	0.3	0.5	0.6	0.8
Ground canola seed	0.0	50.0	100.0	150.0	200.0	250.0		0.0	50.0	100.0	150.0	200.0	250.0
Mineral and vitamin premix ^1^	5.0	5.0	5.0	5.0	5.0	5.0		5.0	5.0	5.0	5.0	5.00	5.0
Total, kg	1.0	1.0	1.0	1.0	1.0	1.0		1.0	1.0	1.0	1.0	1.0	1.0
Calculated composition													
AME, kcal/kg	3150	3151	3153	3153	3154	3152		3200	3200	3200	3200	3200	3200
Dry matter, kcal/kg	884.4	879.9	891.1	894.3	897.4	901.2		886.9	892.2	893.8	901.1	894.9	900.6
Ether extract, g/kg	46.7	40.4	54.1	67.2	80.5	93.9		59.3	63.3	67.9	81.6	83.2	96.5
Crude protein, g/kg	198.0	198.3	198.8	199.3	199.8	198.2		184.0	184.0	184.0	184.1	184.1	184.1
Calcium, g/kg	7.6	8.0	8.0	8.0	8.0	8.0		6.6	6.0	6.0	6.0	6.0	6.0
Phosphorus available, g/kg	3.5	4.7	4.7	4.6	4.6	4.5		4.7	4.7	4.7	4.7	4.8	4.8
Digestible Lys, g/kg	11.3	11.3	11.6	11.4	11.2	10.8		10.6	10.6	10.6	10.6	10.6	10.6
Digestible Met, g/kg	4.5	4.5	4.9	5.2	4.5	4.5		4.2	4.2	4.2	4.2	4.2	4.2
Digestible Met + Cys, g/kg	6.9	6.8	7.1	7.3	6.4	6.3		6.8	6.5	6.4	6.2	6.0	5.9
Digestible Try, g/kg	2.1	2.4	2.2	2.1	2.0	2.1		2.0	2.0	2.0	2.0	1.9	2.0
Digestible Ile, g/kg	7.3	6.8	6.3	5.8	5.3	4.7		6.9	6.4	5.9	5.3	4.8	4.2
Digestible Arg, g/kg	11.6	10.8	10.0	9.2	8.5	7.6		10.9	10.1	9.2	8.3	7.5	6.6
Digestible Thr, g/kg	6.5	6.1	5.6	5.2	4.8	4.3		6.3	5.9	5.4	4.9	4.5	4.0
DEB, mEq/kg	168	150	130	115	101	87		169	150	130	111	93	76
Neutral detergent fiber, g/kg	129.2	128.8	131.7	137.2	142.7	146.1		127.3	130.2	133.1	136.0	140.9	146.4
Acid detergent fiber, g/kg	48.9	51.9	55.2	59.5	63.9	67.5		43.3	46.5	49.8	53.0	57.0	61.4

AME—apparent metabolizable energy; DEB—dietary electrolyte balance (DEB = K + Na − Cl). ^1^ Composition of mineral and vitamin premix (levels per kg of product): Iodine—333.00 mg, Vitamin B12—2133.00 mcg, Manganese—22,400.00 mg, Vitamin K3—320.00 mg, Zinc—17,300.00 mg, Vitamin B6—442.00 mg, BHT—0.80 g, Copper—2800.00 mg, Vitamin E—2830.00 UI Niacin—5330.00 mg, Vitamin D3—454,000.00 IU, Choline—58.07 g, Iron—17,000.00 mg, Vitamin B1—357.00 mg, Selenium—100.00 mg, Vitamin A—1,880,000.00 IU, Vitamin B2—800.00 mg, Panthenic Acid—2086.70 mg.

**Table 5:** Values of the AME (kcal/kg), AMEn (kcal/kg), MCAME (%), MCAMEn (%), MCEE (%), MCDM (%), and MCCP (%) for ground and whole canola seed, with or without the enzyme.

Item		AME	AMEn	MCAME	MCAMEn	MCEE	MCDM	MCCP
Seed	Ground	4182	4049	65.5	63.4	72.9	73.2	67.3
	Whole	3000	2872	46.9	44.9	56.9	72.5	66.1
Enzyme	With	4091	3958	64.1	61.9	66.3	73.3	67.8
	Without	3091	2963	48.4	46.4	63.5	72.3	65.4
Level	100 g/kg	3816	3708	47.1	45.0	70.1	77.5	67.9
	200 g/kg	3477	3321	43.5	42.4	62.7	77.4	65.7
CV (%)		11.69	13.21	3.21	3.44	7.60	2.74	5.97
*p*-Value								
Seed		<0.001	<0.001	<0.001	<0.001	<0.001	0.320	0.390
Enzyme		<0.001	<0.001	<0.001	<0.001	0.080	0.120	0.070
Level			0.005	0.004	0.015	0.040	<0.001	0.090
Seed × Enzyme			<0.001	<0.001	0.001	0.001	0.260	0.760
Seed × Level			0.018	0.011	0.001	0.002	0.003	0.980
Enzyme × Level			0.313	0.628	0.830	0.770	0.300	0.530
Seed × Enzyme × Level			0.112	0.164	0.660	0.160	0.060	0.630

AME—apparent metabolizable energy, AMEn—AME corrected for nitrogen, CV—coefficient of variation, MCAME—metabolizable coefficient of AME, MCAMEn—metabolizable coefficient of AMEn, MCEE—metabolizable coefficient of ether extract, MCDM—metabolizable coefficients of dry matter, MCDP—metabolizable coefficient of crude protein.

**Table 6:** Unfolding of the interaction for the AME, AMEn, MCAME, and MCAMEn of whole and ground canola seeds, with or without the enzyme, and between substitution levels of 100 and 200 g/kg.

			**AME (kcal/kg)**	**AMEn (kcal/kg)**	
**Item**		**GCS**	**WCS**	**GCS**	**WCS**
Enzyme	Without	3864 Ba	2318 Bb	3734 Ba	2231 Bb
	With	4500 Aa	3906 Ab	4306 Aa	3783 Ab
Level	100 g/kg	4210 Aa	3423 Ab	4050 Aa	3366 Ab
	200 g/kg	4154 Aa	2800 Bb	3996 Aa	2647 Bb
		**MCAME (%)**		**MCAMEn (%)**	
**Item**		**GCS**	**WCS**	**GCS**	**WCS**
Enzyme	Without	60.5 Ba	36.3 Bb	58.5 Ba	34.9 Bb
	With	70.5 Aa	61.2 Ab	67.4 Aa	59.3 Ab
Level	100 g/kg	65.9 Aa	53.6 Ab	63.4 Aa	52.7 Ab
	200 g/kg	65.1 Aa	43.9 Bb	62.6 Aa	41.5 Bb

AME—apparent metabolizable energy; AMEn—AME corrected for nitrogen; GCS—ground canola seed; MCAME—metabolizable coefficient of AME; MCAMEn—metabolizable coefficient of AMEn; WCS—whole canola seed; (A-B)—differs statistically in the column; (a-b)—differs statistically in the rows. The means in each column (A-B) or each row (a-b) for each item differ significantly by Tukey’s test (*p* < 0.05).

**Table 7:** Metabolizable coefficients of apparent metabolizable energy (MCAMEs) and corrected for nitrogen (MCAMEn) of experimental diets.

Item		MCAME (%)	MCAMEn (%)
Seed	Ground	78.1	77.8
	Whole	76.5	75.1
Enzyme	With	77.5	77.1
	Without	76.1	75.7
*p*-Value			
Seed		<0.001	<0.001
Enzyme		0.001	0.001
Enzyme × Seed		0.962	0.938

**Table 8:** Effects of the inclusion of ground canola seeds in the diet of broilers from 1 to 42 days of age on growth performance.

Item	Inclusion Level of Ground Canola Seed (g/kg)						SEM	*p*-Value
	0	50	100	150	200	250		
1–7 days								
FI	19.4	20.6	19.2	19.6	19.7	20.3	0.23	0.490
BW	153.0	153.0	147.2	143.5 *	143.9 *	140.8 *	1.17	0.001
BWG	15.2	15.0	14.3	13.8 *	13.8 *	13.3 *	0.16	0.003
FCR	1.28	1.37	1.34	1.42	1.43 *	1.53 *	0.03	0.003
7–14 days								
FI	40.1	41.8	39.8	42.2	40.0	41.5	0.46	0.560
BW	354.9	356.0	339.8	328.1 *	326.5 *	322.3 *	3.58	0.005
BWG	28.8	29.0	27.5	26.4	26.1	25.9 *	0.38	0.040
FCR	1.39	1.44	1.45	1.61	1.54	1.61	0.03	0.090
14–21 days								
FI	66.9	68.3	66.4	70.4	67.2	68.9	0.86	0.800
BW	747.9	755.0	716.7	711.1	675.2 *	666.5 *	8.52	0.002
BWG	56.2	57.0	53.8	54.7	49.8	49.2 *	0.82	0.010
FCR	1.21	1.20	1.23	1.30	1.36	1.41	0.03	0.190
21–28 days								
FI	104.0	108.7	102.3	108.9	102.4	104.8	1.52	0.700
BW	1215.0	1218.0	1166.5	1166.4	1095.2 *	1088.3 *	14.55	0.014
BWG	64.5	66.9	65.3	61.0	65.2	59.5	1.36	0.630
FCR	1.61	1.63	1.57	1.79	1.60	1.78	0.03	0.110
28–35 days								
FI	138.0	146.2	143.7	145.1	140.6	143.2	1.46	0.650
BW	1861.3	1836.0	1796.0	1770.9	1707.9 *	1578.1 *	19.69	<0.001
BWG	80.6	86.2	87.4	90.2	81.7	88.3	1.86	0.660
FCR	1.72	1.70	1.65	1.62	1.82	1.63	0.04	0.790
35–42 days								
FI	166.4	169.7	171.2	170.5	167.2	175.6	1.72	0.720
BW	2463.0	2468.3	2405.1	2350.2	2273.6 *	2125.5 *	26.07	<0.001
BWG	83.2	81.4	86.2	83.5	80.8	90.1	1.41	0.440
FCR	2.00	2.09	2.00	2.05	2.08	1.95	0.02	0.250
1–42 days								
FI	89.1	92.6	90.4	92.8	89.5	92.4	0.85	0.583
CFI	3742.2	3887.1	3798.1	3897.2	3759.8	3879.9	33.34	0.583
BWG	57.5	57.6	56.2	54.8	53.0 *	49.5 *	0.36	<0.001
FCR	1.55	1.61	1.61	1.70	1.70	1.87 *	0.02	<0.001

BW—body weight, g; BWG—body weight gain, g/day; CFI—cumulative feed intake, g; FCR—feed conversion ratio; FI—average daily feed intake, g; SEM—standard error of the mean. * The means in each column for each item differ significantly from control by Dunnett’s test (*p* < 0.05).

**Table 9:** Effects of the inclusion of ground canola seeds in the diet of broilers from 1 to 42 days of age on carcass yield and commercial cuts.

Item	Inclusion Level of Ground Canola Seed (g/kg)						SEM	*p*-Value
	0	50	100	150	200	250		
Carcass yield	74.2	74.6	74.9	75.5	73.9	73.0	1.96	0.470
Breast yield	33.5	34.2	34.7	35.3	34.2	33.5	1.27	0.200
Thigh and overcoat yield	30.5	29.5	29.9	29.5	29.1	30.1	0.67	0.300
Wing yield	11.2	10.9	11.3	11.2	11.6	11.7	0.39	0.300
Back yield	24.1	24.4	23.5	23.0	24.3	24.1	0.74	0.050

SEM—standard error of the mean.

**Table 10:** Effects of the inclusion of ground canola seeds in the diet of broilers from 1 to 42 days of age on the serum biochemical profile.

Item	Inclusion Level of Ground Canola Seed (g/kg)						SEM	*p*-Value
	0	50	100	150	200	250		
Uric acid	2.36	2.21	1.91	1.85	2.62	1.86	0.49	0.100
Total proteins	2.86	2.85	2.79	2.76	2.82	2.72	0.22	0.920
Serum calcium	4.51	4.89	4.82	4.98	4.75	4.32	0.56	0.450
Chloride	104.5	105.2	104.7	104.5	105.6	105.9	2.17	0.860
Cholesterol	149.6	152.2	148.9	154.4	158.6	149.2	19.43	0.960
Alkaline phosphatase	576.3	634.3	1085.9	1082.9	947.5	1023.7	445.08	0.220
Total serum phosphorus	5.67	6.18	5.73	5.89	5.90	6.10	0.58	0.720
Triglycerides	56.3	59.2	50.8	52.1	48.0	52.0	11.12	0.660

SEM—standard error of the mean.

**Table 11:** Effects of the inclusion of ground canola seeds in the diet of broilers from 1 to 42 days of age on the organ index.

Item	Inclusion Level of Ground Canola Seed (g/kg)						SEM	*p*-Value
	0	50	100	150	200	250		
Gizzard index	1.78	1.83	1.82	1.80	1.94	2.14	0.22	0.140
Liver index	2.01	1.96	2.08	1.97	2.05	2.07	0.16	0.790
Spleen index	0.10	0.08	0.10	0.09	0.07	0.09	0.02	0.190
Heart index	0.57	0.58	0.58	0.57	0.55	0.59	0.08	0.980
Intestine index	4.58	4.18	4.46	3.96	4.48	4.52	0.42	0.200

SEM—standard error of the mean.

## Data Availability

The original contributions presented in the study are included in the article; further inquiries can be directed to the first author.

## Abbreviations

The following abbreviations are used in this manuscript:

GCS	Ground canola seed
AME	Apparent metabolizable energy
AMEn	Apparent metabolizable energy corrected for nitrogen retention
FCR	Feed conversion ratio
BW	Body weight
BWG	Body weight gain
EE	Ether extract
GE	Gross energy
NSPs	Non-starch polysaccharides
DM	Dry matter
CP	Crude protein
NDF	Neutral detergent fiber
ADF	Acid detergent acid
RD	Reference diet
WCS	Whole canola seed
GMD	Geometric mean diameter
FI	Feed intake
MCDM	Metabolizable coefficient of DM
MCCP	Metabolizable coefficient of CP
MCEE	Metabolizable coefficient of EE
MCAME	Metabolizable coefficient of AME
MCAMEn	Metabolizable coefficient of AMEn

## References

[1] Toghyani M., Swick R.A., Barekatain R. (2017). Effect of Seed Source and Pelleting Temperature during Steam Pelleting on Apparent Metabolizable Energy Value of Full-Fat Canola Seed for Broiler Chickens. Poult. Sci..

[2] Ibrahim N., Sabic E., Abu-Taleb A., Abdel-Moneim A. (2020). Effect of Dietary Supplementation of Full-Fat Canola Seeds on Productive Performance, Blood Metabolites and Antioxidant Status of Laying Japanese Quails. Braz. J. Poult. Sci..

[3] Abdel-Moneim A.-M.E., Sabic E.M., Abu-Taleb A.M., Ibrahim N.S. (2020). Growth Performance, Hemato-Biochemical Indices, Thyroid Activity, Antioxidant Status, and Immune Response of Growing Japanese Quail Fed Diet with Full-Fat Canola Seeds. Trop. Anim. Health Prod..

[4] Kandel M., Macelline S.P., Toghyani M., Selle P.H., Liu S.Y. (2025). The Impact of Canola Meal and Canola Seed Inclusions in Broiler Diets. Poult. Sci..

[5] Meng X., Slominski B.A., Nyachoti C.M., Campbell L.D., Guenter W. (2005). Degradation of Cell Wall Polysaccharides by Combinations of Carbohydrase Enzymes and Their Effect on Nutrient Utilization and Broiler Chicken Performance. Poult. Sci..

[6] Jia W., Slominski B.A., Guenter W., Humphreys A., Jones O. (2008). The Effect of Enzyme Supplementation on Egg Production Parameters and Omega-3 Fatty Acid Deposition in Laying Hens Fed Flaxseed and Canola Seed. Poult. Sci..

[7] Thanabalan A., Mohammadigheisar M., Kiarie E.G. (2021). Amino Acids and Energy Digestibility in Extruded or Roasted Full Fat Soybean Fed to Broiler Chickens without or with Multienzyme Supplement Containing Protease, Phytase, and Fiber Degrading Enzymes. Poult. Sci..

[8] González-Alvarado J.M., Jiménez-Moreno E., González-Sánchez D., Lázaro R., Mateos G.G. (2010). Effect of Inclusion of Oat Hulls and Sugar Beet Pulp in the Diet on Productive Performance and Digestive Traits of Broilers from 1 to 42 Days of Age. Anim. Feed. Sci. Technol..

[9] Barekatain M.R., Wu S.B., Toghyani M., Swick R.A. (2015). Effects of Grinding and Pelleting Condition on Efficiency of Full-Fat Canola Seed for Replacing Supplemental Oil in Broiler Chicken Diets. Anim. Feed. Sci. Technol..

[10] Hamadi S., Salari S., Aghaei A., Ghorbani M.R. (2023). Changes in Performance and Apparent Ileal Digestibility of Broiler Chickens Fed Diets Containing Electron-Irradiated Full-Fat Canola Seed. Radiat. Phys. Chem..

[11] Eratak S., Cabuk M. (2019). Effect of the Dietary Inclusion of Full Fat Canola Seed on Performance of Quails. Int. J. Sci. Res. Manag..

[12] Brand T.S., De Brabander L., Van Schalkwyk S.J., Pfister B., Hays P. (2000). The True Metabolisable Energy Content of Canola Oilcake Meal and Full-Fat Canola Seed for Ostriches (*Struthio camelus*). Br. Poult. Sci..

[13] Rutkowski A., Kaczmarek S., Szczyrkowska A., Józefiak D. (2012). The Effect of Particle Size of Full-Fat Rapeseed and of Multi-Carbohydrase Enzyme Supplementation on Nutrient Digestibility and Performance in Broilers. J. Anim. Feed. Sci..

[14] Rostagno H.S., Albino L.F.T., Hannas M.I., Campos G.P., Lopes T.C., Vieira R.A., Freitas C.D.P., Oliveira B.C., Saraiva B.V.R., Brito R.P. (2017). Tabelas Brasileiras Para Aves e Suínos: Composição de Alimentos e Exigências Nutricionais.

[15] Brazilian Agricultural Research Corporation (2013). *Embrapa Swine and Poultry Granucalc: Software for Calculating Geometric Mean Diameter (GMD) and Geometric Standard Deviation (GSD)*.

[16] Latimer G.W. (2023). Official Methods of Analysis of AOAC International.

[17] Potter L.M., Matterson L.D. (1960). Metabolizable Energy of Feed Ingredients for the Growing Chick. Poult. Sci..

[18] Hill F.W., Anderson D.L. (1958). Comparison of Metabolizable Energy and Productive Energy Determinations with Growing Chicks. J. Nutr..

[19] Slominski B.A., Meng X., Campbell L.D., Guenter W., Jones O. (2006). The Use of Enzyme Technology for Improved Energy Utilization from Full-Fat Oilseeds. Part II: Flaxseed. Poult. Sci..

[20] Suyama M.S., Pinheiro M.J., Rosa L.L., Ferreira L.M.O., Gewehr C.E. (2025). Energetic Value and Digestibility of Kikuyu Grass and Annual Ryegrass with the Inclusion of Exogenous Enzymes for Laying Hens. Poult. Sci..

[21] Barbour G.W., Sim J.S. (1991). True Metabolizable Energy and True Amino Acid Availability in Canola and Flax Products for Poultry. Poult. Sci..

[22] Mateos G.G., Jiménez-Moreno E., Serrano M.P., Lázaro R.P. (2012). Poultry Response to High Levels of Dietary Fiber Sources Varying in Physical and Chemical Characteristics. J. Appl. Poult. Res..

[23] Zhang F., Adeola O. (2017). Energy Values of Canola Meal, Cottonseed Meal, Bakery Meal, and Peanut Flour Meal for Broiler Chickens Determined Using the Regression Method. Poult. Sci..

[24] Assadi E., Janmohammadi H., Taghizadeh A., Alijani S. (2011). Nutrient Composition of Different Varieties of Full-Fat Canola Seed and Nitrogen-Corrected True Metabolizable Energy of Full-Fat Canola Seed with or without Enzyme Addition and Thermal Processing. J. Appl. Poult. Res..

[25] Barekatain R., Swick R.A., Toghyani M., de Koning C.T. (2017). Interactions of Full-Fat Canola Seed, Oat Hulls as an Insoluble Fiber Source and Pellet Temperature for Nutrient Utilization and Growth Performance of Broiler Chickens. Poult. Sci..

[26] Tripathi M.K., Mishra A.S. (2007). Glucosinolates in Animal Nutrition: A Review. Anim. Feed. Sci. Technol..

[27] Mateos G.G., Cámara L., Fondevila G., Lázaro R.P. (2019). Critical Review of the Procedures Used for Estimation of the Energy Content of Diets and Ingredients in Poultry. J. Appl. Poult. Res..

[28] Nir I., Nitsan Z., Mahagna M. (1993). Comparative Growth and Development of the Digestive Organs and of Some Enzymes in Broiler and Egg Type Chicks after Hatching. Br. Poult. Sci..

[29] Lee K.-H., Olomu J.M., Sim J.S. (1991). Live Performance, Carcass Yield, Protein and Energy Retention of Broiler Chickens Fed Canola and Flax Full-Fat Seeds and the Restored Mixtures of Meal and Oil. Can. J. Anim. Sci..

[30] Józefiak D., Ptak A., Kaczmarek S., Maćkowiak P., Sassek M., Slominski B.A. (2010). Multi-Carbohydrase and Phytase Supplementation Improves Growth Performance and Liver Insulin Receptor Sensitivity in Broiler Chickens Fed Diets Containing Full-Fat Rapeseed. Poult. Sci..

